# Herpes simplex virus 1 glycoprotein C promotes virus penetration from endosomes during entry, independent of interaction with heparan sulfate

**DOI:** 10.3389/fmicb.2025.1549349

**Published:** 2025-04-09

**Authors:** Seth M. Schneider, Giulia Tebaldi, Katrina A. Gianopulos, Darin J. Weed, Suzanne M. Pritchard, Chloe Leach, Anthony V. Nicola

**Affiliations:** ^1^Department of Veterinary Microbiology and Pathology, College of Veterinary Medicine, Washington State University, Pullman, WA, United States; ^2^School of Molecular Biosciences, College of Veterinary Medicine, Washington State University, Pullman, WA, United States

**Keywords:** herpes simplex virus, fusion, glycoprotein C, heparan sulfate, virus entry, virus penetration

## Abstract

Herpes simplex virus 1 (HSV-1) gC is a multi-functional glycoprotein present in the viral envelope and on the surface of infected cells. Virion gC, and to a lesser extent the fusion protein gB, interacts with host heparan sulfate to mediate HSV-1 attachment to the cell surface. Virion gC selectively facilitates HSV-1 entry into cells that support entry by a low pH-dependent endocytic pathway. gC regulates fusion-associated conformational changes in gB. Here we investigated the mechanism by which gC plays a post-attachment role in HSV-1 entry into cells. HSV-1 entered HS-deficient cells by a low pH-dependent route. Similarly, HSV-1 deleted for HS-binding domains entered HS-bearing cells by a low pH pathway. Thus, the presence of HS on cells and the ability of HSV-1 to engage HS do not direct HSV-1 to a pH-dependent entry pathway. HSV-1 lacking gC accumulated in endosomes during viral entry, supporting the notion that gC influences viral penetration from endosomes. Interestingly, the pH-neutral cell–cell fusion mediated by HSV-1 glycoproteins gB, gD, and gH/gL was not altered by gC. Soluble heparin inhibited cell–cell fusion regardless of the presence of gC or heparan sulfate. The kinetics of endocytic uptake of gC-null HSV-1 was rapid and very similar to wild type virus. Thus, the role of gC in regulating low pH entry of HSV-1 occurs downstream of internalization of enveloped particles from the plasma membrane. Together, the results presented here and elsewhere support a post-attachment, post-internalization function for gC in HSV-1 entry that is independent of HS.

## Introduction

Herpes simplex virus (HSV) infections are ubiquitous in the human population and cause significant morbidity and mortality. HSV-1 can enter cells by multiple cellular pathways. HSV-1 enters many cell types, including human keratinocytes and model CHO cells, by endocytosis. This is a low pH-dependent pathway, as viral penetration requires the mildly acid pH of host endocytic compartments (Nicola et al., 2005; Fuller and Spear, 1987; Koyama and Uchida, 1987; Nicola et al., 2003; Nicola, 2016). HSV-1 enters other cells, such as human neurons or model Vero cells, by direct penetration at the plasma membrane of the host cell. This entry route occurs at physiologic pH, and is often called a pH-neutral or pH-independent pathway. Regardless of the entry pathway utilized, fusion of the HSV-1 membrane with a cellular membrane requires the coordinated action of viral envelope glycoproteins B, D, and the heterodimer H/L (gB, gD, gH, gL) (Weed and Nicola, 2017; Connolly et al., 2020; Madavaraju et al., 2020; Gonzalez-Del Pino and Heldwein, 2022). The molecular details of HSV-1 fusion and entry and how HSV-1 chooses an entry pathway are incompletely understood. We demonstrated that HSV-1 glycoprotein C (gC) impacts viral entry into cells that support a low pH endosomal entry route for HSV-1 (Komala Sari et al., 2020c). gC is the only HSV-1 protein currently known to function selectively in endosomal entry of HSV-1. Envelope gC regulates the conformational changes in the fusion protein gB (Komala Sari et al., 2020c). In the absence of gC, HSV-1 exhibits a delay in penetration from the endosome and fusion-associated conformational changes in gB require an even lower pH. This novel function of gC is independent of its role in virion attachment to the cell surface (Komala Sari et al., 2020c). gC itself undergoes pH-triggered conformational changes, and a gC monoclonal antibody selectively inhibits low pH entry of HSV-1 (Gianopulos et al., 2022). The precise mechanism of how gC facilitates HSV-1 entry by a low pH entry pathway remains elusive and is the subject of this work.

In most models of HSV-1 entry, the first step is attachment of virions to the host cell via gC binding to cell surface heparan sulfate (HS) (Shukla and Spear, 2001). gB, to a lesser extent, also binds HS (Herold et al., 1994; Williams and Straus, 1997; Laquerre et al., 1998). Cell surface HS is not absolutely required for viral attachment or membrane fusion. The influence of gC on virus-cell fusion during entry was suggested previously (Komala Sari et al., 2020c), and here we interrogate gC’s role in fusion with the established virus-free assay for cell–cell fusion by transiently expressing the HSV-1 entry glycoproteins on the surface of effector cells. Soluble heparin inhibits cell–cell fusion mediated by gB, gD, and gH/gL, but the mechanism of this inhibition is not completely understood (Foster et al., 2001). Given that gB and gC both bind to HS and may functionally interact during endosomal entry, we interrogate the impact of gC or HS in several processes, including virion internalization from the cell surface, cell–cell fusion, the pH-dependence of viral entry, and the ability of soluble heparin to inhibit cell–cell fusion. This report fills the following gaps in our understanding of the impact of gC and its binding partner heparan sulfate in endocytic entry of HSV-1. (i) The basolateral nature of entry of HSV-1 into polarized cells is independent of gC. (ii) HSV-1 particles lacking gC accumulate in endosomes as detected by electron microscopy (EM). (iii) Cell surface heparan sulfate does not direct HSV-1 to a low pH-dependent entry pathway. (iv) The ability of HSV-1 to bind to heparan sulfate does not determine HSV-1’s selection of a low pH entry pathway. (v) The kinetics of endocytic uptake of HSV-1 lacking gC are rapid and very similar to wild type. (vi) Wild type gC does not impact cell–cell fusion mediated by wild type gB, gD, and gH/gL. (vii) The HSV-1 strain ANG allele of gC does not impact cell–cell fusion mediated by the ANG alleles of gB, gD, and gH/gL. (viii) Heparin inhibition of nectin-1-mediated cell–cell fusion in the presence of gC is independent of cell surface heparan sulfate. In total, we provide evidence that HSV-1 particles are internalized from the plasma membrane in a gC-independent manner and that gC-null virions accumulate in endosomes, consistent with a low pH penetration defect.

## Materials and methods

### Cells and viruses

Vero cells and HaCaT keratinocytes were propagated in Dulbecco’s modified Eagle’s medium (DMEM; Thermo Fisher Scientific) supplemented with 10% fetal bovine serum (FBS; Atlanta Biologicals). CHOpgs745A cells (ATCC), which lack a gene required for heparan sulfate biosynthesis, and Chinese hamster ovary (CHO)-K1 cells were propagated in Ham’s F-12 nutrient mixture supplemented with 10% FBS (Atlanta Biologicals). CHO-HVEM (M1A) (Montgomery et al., 1996) and CHO-nectin-2 (M2A) (Warner et al., 1998) cells were provided by Drs. Roselyn Eisenberg and Gary Cohen (University of Pennsylvania) and were propagated in Ham’s F12 medium supplemented with 10% FBS and pen-strep glutamine. CHO-HVEM or CHO-nectin-2 cells are stably transformed with genes for human HVEM or nectin-2 respectively, and the *Escherichia coli lacZ* gene under the control of the HSV-1 ICP4 promoter. CHO-HVEM and CHO-nectin-2 cells were selected by propagation in Ham’s F12 medium supplemented with puromycin (Sigma) and G418 (Thermo Fisher Scientific). Cells were propagated in non-selective medium prior to use in experiments.

Wild type HSV-1 strain KOS was obtained from Priscilla Schaffer (Harvard University). HSV-1 (KOS) ∆gC2-3 (or HSV-1 ∆gC) contains the *lacZ* gene in place of most of the gC gene and is considered gC-null (Herold et al., 1994). HSV-1 (KOS) gC2-3Rev (or HSV-1 gCR) is the rescuant virus containing the wild type gC gene (Herold et al., 1994). These viruses were kindly provided by Curtis Brandt, University of Wisconsin-Madison. The recombinant viruses HSV-1 gBpK^−^gCR, HSV-1 gBpKRgC^−^, and HSV-1 gBpK^−^gC^−^ were kindly provided by Joseph Glorioso (University of Pittsburgh). These viruses are either null or rescued for the HS-binding polylysine (pK) region of gB and/or gC (Laquerre et al., 1998). The HSV-1 strains used in this study were propagated and titered on Vero cells.

### Immunofluorescence microscopy

For infection of polarized cells, HaCaT cells were seeded at low density on round glass coverslips in 24-well plates. Islet monolayers were formed by 4 days of culture. Cultures were mock-treated or treated with 50 mM ammonium chloride for 20 min at 37°C and then infected with HSV-1 KOS ΔgC or gCR (1.1 × 10^7^ PFU/coverslip; MOI < 1) for 4 h in the presence of drug. Low MOI infection allows detection of entry via the preferred surfaces (basolateral) in the polarized cultures. Higher MOI results in all cells infected (Nicola et al., 2005). HaCaT islets were fixed with ice-cold methanol and stained with ICP4 monoclonal antibody (P1101, Virusys), followed by anti-mouse Alexa Fluor 647 (Thermo Fisher Scientific, Waltham, MA), and counterstained with 4,6–diamidino-2-phenylindole dihydrochloride (DAPI; Roche). For heparinase experiments, CHO-HVEM cells were mock-treated or treated with heparinase II (Sigma, St. Louis, MO) and heparinase III (Sigma) at 37°C for 2 h. Following HSV-1 infection, cells were fixed with 100% methanol. Samples were stained with 10E4 antibody to HS (Amsbio, Cambridge, MA), followed by anti-mouse Alexa Fluor 488 (Thermo Fisher Scientific), and counterstained with DAPI. Images were obtained with a Leica DMi8 microscope and processed using Adobe Photoshop CS5.1.

### Transmission electron microscopy

HSV-1 ΔgC or gCR (MOI of 150–500) was added to cells (MOI of 150–500) for 30 min at 37°C. These very high MOIs are necessary to visualize relatively rare events by EM. Infected cells were washed with PBS, scraped, pelleted, and then fixed in 0.1 M phosphate buffer containing 2% paraformaldehyde, 2% glutaraldehyde (pH 7.2) overnight at 4°C. The cell pellet was then microwave fixed (2.5 min, 250 W, 28°C cutoff temperature) and incubated at room temperature for 5 min. Pellet was washed three times in 0.1 M phosphate buffer and then fixed in 1% osmium tetraoxide for 1 h at room temperature or 2% osmium tetraoxide overnight at 4°C. The cell pellet was washed three times in water and then dehydrated in graded ethanol (30, 50, 70, 95, and 100%), followed by 100% propylene oxide, and infiltrated in a 1:1 mixture of propylene oxide and SPURRS resin for 1 h before 100% SPURRS resin overnight. Resin was changed and sample was cured at 60°C overnight. The cured block was thin-sectioned and stained in 2% aqueous uranyl acetate and Reynold’s lead (Electron Microscopy Sciences, Hatfield, PA, United States). Samples were visualized with a Tecnai G2 20 Twin transmission electron microscope (Field Emission Instruments Company, Hillsboro, OR, United States) at 40 kV. Images were captured with a 4 K Eagle digital camera and processed using Adobe Photoshop CS5.1. To determine the relative amounts of HSV-1 ∆gC or gCR in endosomes at 30 min post-infection (p.i.), electron micrograph (EM) negatives of infected, CHO-HVEM cells were taken in a systematic random fashion for each sample. Approximately 30 to 60 intracellular, membrane-bound vesicles containing a total of approximately 110 enveloped virions were evaluated per sample. The percentage of endosomes containing at least two HSV-1 particles was calculated.

### β-Galactosidase reporter assay for HSV-1 entry

CHO-HVEM cells in quadruplicate were pretreated with increasing concentrations of ammonium chloride for 20 min at 37°C. HSV-1 KOS, gBpK^−^gCR, gBpKRgC^−^, or gBpK^−^gC^−^ (MOI 0.2–1) was added to cells at 37°C for 6 h in the continued presence of ammonium chloride. These MOIs are necessary to obtain a robust β-galactosidase activity reading within the linear range of detection. Cells were lysed in 0.5% IGEPAL (Sigma), and chlorophenol red-β-D-galactopyranoside substrate for β-galactosidase (Roche Diagnostics, Indianapolis, IN) was added. β-galactosidase activity was read at 595 nm in a BioTek microplate reader as an indicator of viral entry.

### Internalization assay

Confluent CHO-HVEM cell monolayers were washed once with ice-cold PBS. Ice-cold carbonate-free, serum-free Ham’s F12 medium supplemented with 20 mM HEPES and 0.2% bovine serum albumin (binding medium) was added to cultures for 10 min at 4°C on ice. HSV-1 KOS was bound to cells for 2 h at 4°C on ice (MOI of 0.5) in binding medium. Low MOI infection facilitates experimental detection of virion internalization that is <100%. Cells were washed with PBS and incubated at 37°C in complete Ham’s F12 medium. Extracellular virus was inactivated by 5 min room temperature sodium citrate (pH 3.0) treatment at the indicated times. At 8 h p.i. cells were fixed with ice-cold methanol, and random fields of 400 to 500 cells per sample were evaluated. Total cell number was enumerated by nuclear staining with DAPI. Infected cells were detected by immunofluorescence with anti-HSV-1 ICP4 mouse monoclonal antibody P1101 (Virusys) followed by goat anti-mouse IgG labeled with Alexa Fluor 488 (Invitrogen). Cells were imaged with a Leica DMi8 fluorescence microscope. Maximum infectivity was set to 100%.

### Plasmids

DNA plasmids pCAGGS (empty vector) and those encoding HSV-1 strain KOS gB (pPEP98), gD (pPEP99), gH (pPEP100), gL (pPEP101) (Pertel et al., 2001), T7 RNA polymerase (pCAGT7), and luciferase enzyme (pT7EMCLuc) were kindly provided by Patricia Spear, Northwestern University. Plasmid pSH140 encoding KOS gC was kindly provided by Gary Cohen and Roz Eisenberg, University of Pennsylvania (Hung et al., 1992). Plasmids encoding HSV-1 strain ANG gB (pSP1), gC (pSP3), gD (pSP4), gH (pSP5), and gL (pSP2) were previously described (Gianopulos et al., 2024).

### Virus-free reporter assay for cell–cell fusion mediated by HSV-1 glycoproteins

Confluent CHO-K1 effector cells in 6-well plates were transfected with 0.5 μg each of the indicated plasmids. A total of 3–4 μg DNA was added per well of effector cells. Confluent CHO-K1 target cells in 6-well plates were transfected with 7.5 μg each of the indicated plasmids per plate. Transfections were performed by adding p3000 reagent to DNA in OptiMEM at a ratio of 2 μL:1 μg DNA. This mixture was added to Lipofectamine 3000 at a ratio of 2 μL Lipofectamine 3,000:1 μg DNA. Samples were mixed and incubated at room temperature for 20 min. Cells were washed once with PBS, and samples were added to the cells. At 6 h post-transfection, cells were washed once with PBS and trypsinized. Target cells were added to effector cells at a 1:1 ratio, and cells were replated in triplicate onto 24-well plates. Cells were co-cultured for 18 h at 37°C. Cells were then lysed and subjected to one freeze–thaw cycle at −80°C. Samples were transferred to dark 96-well plates, and luciferase substrate was added. Luminescence was detected with a BioTek Synergy Neo luminometer. Stocks of 0.5 mg/mL heparin (Sigma) were prepared in water and stored at −20°C.

### Statistical analysis

All experiments were independently performed a minimum of three times. Data and statistical analyses were performed with Excel (Microsoft) and GraphPad Prism 9.0 software. For direct pairwise comparisons in microscopy, entry, and fusion experiments, standard two-tailed Student’s *t*-tests were implemented. The assumptions of the *t*-test are normal distribution of data, appropriate sample size, and a homogeneous variance. Error bars indicate standard errors between experiments. *p* values represent the following: *p < 0.05; **p < 0.01; and ***p < 0.001.

## Results

### Role of gC in directing HSV-1 to infect basolateral surfaces of polarized human keratinocytes

**Figure 1:**
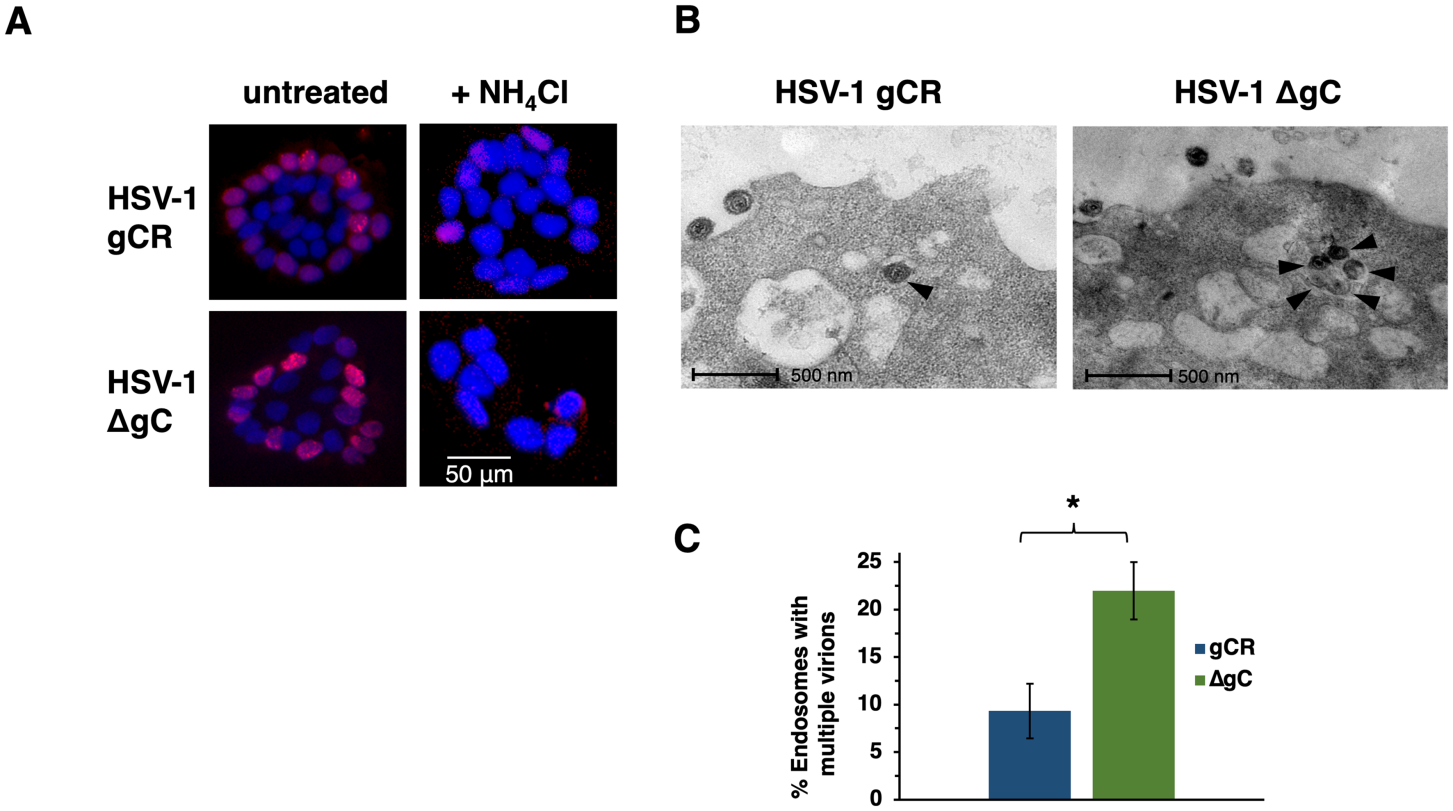
Impact of gC on the basolateral route of HSV-1 infection of polarized cells and on accumulation of entering HSV-1 particles in endosomes. **(A)** HaCaT cells were seeded at low density on glass coverslips. Islets formed by 4 days of culture. Cultures were left untreated or treated with 50 mM ammonium chloride for 20 min at 37°C, and were infected with HSV-1 ΔgC or gCR (MOI ~ 0.5) for 4 h. Cultures were fixed and stained with MAb P1101 to ICP4 (red), and counterstained with DAPI. Images are representative of at least three independent experiments. **(B)** HSV-1 ∆gC or gCR was added to CHO-HVEM cells (MOI 150–500). At 30 min. p.i., cells were fixed and processed for TEM analysis. Representative images are shown. Black arrowheads indicate intracellular, enveloped HSV particles. **(C)** Endosomes containing at least two virions were enumerated. *p-value < 0.05, Student’s *t* test.

HSV-1 gC has multiple functions in HSV-1 entry. A previous report suggested that gC determined the polarity of HSV-1 entry into Madin Darby canine kidney cells (Sears et al., 1991). HSV-1 preferentially enters polarized HaCaT islet cultures via the basolateral surfaces (Nicola et al., 2005). HSV-1 ∆gC has a ~ 1 log defect in plaquing efficiency relative to wild type on HaCaT monolayer cultures, which support endosomal entry of HSV-1 (Herold et al., 1991; Komala Sari et al., 2020c; Nicola et al., 2005). Since gC promotes preferential entry into epithelial cells by endocytosis (Komala Sari et al., 2020c), we determined whether gC impacts the basolateral route of entry into polarized human HaCaT keratinocytes. Cells toward the center of islets have their apical surfaces exposed and their basolateral surfaces are less accessible. At MOI ~ 0.5, HSV-1 ∆gC and the wild-type-equivalent virus gCR infected keratinocytes via basolateral surfaces (Figure 1A). Under these experimental conditions, little to no apical infection of HaCaT cells was detected. We next probed whether gC influenced the low pH-dependence of basolateral entry. HSV-1 ∆gC or HSV-1 gCR was added to islets in the presence of ammonium chloride, which elevates the normally low pH of endosomes and inhibits HSV-1 entry into human epithelial cells (Nicola, 2016; Nicola et al., 2003). Ammonium chloride inhibited infection of polarized HaCaT cultures by both HSV-1 ∆gC and gCR, suggesting that gC is not required for the pH-dependence of entry from basolateral surfaces (Figure 1A). In apparent contrast to a previous report (Sears et al., 1991), the results here suggest that the basolateral nature of entry of HSV-1 into polarized cells is independent of gC.

### HSV-1 lacking gC accumulates in endosomes during viral entry

HSV-1 ∆gC has delayed entry kinetics relative to wild-type. In cells that support endosomal entry of HSV-1, the time course of recovery of infectious enveloped particles from cells during entry suggests delayed penetration of gC-null virus from endocytic compartments (Komala Sari et al., 2020c). We theorized that enveloped gC-null virions persist in endosomal compartments, consistent with a function for gC in penetration from low pH endosomes. The conclusion from the previous paper was inferred from biological experiments. To provide direct visual evidence, HSV-1 virions deleted for gC were added to cells, and then virion-containing endosomes were detected by transmission electron microscopy during viral entry. HSV-1 ∆gC or gCR was added to CHO-HVEM cells, a prototypical model cell line that supports endocytic entry of HSV-1. More is known about HSV-1 entry into CHO cells than any other cell type. At 30 min p.i., vesicles containing increased numbers HSV-1 ∆gC particles were more apparent than wild type HSV-1 gCR (Figure 1B). More vesicles containing at least two ∆gC virions were detected, suggesting accumulation of gC-null HSV-1 in endosomal compartments (Figure 1C). These results visually support the defective entry and lag in endosomal penetration observed previously in biological assays. Altogether, results suggest that gC facilitates HSV-1 penetration from endosomes.

### HSV-1 enters HS-deficient cells by a low pH-dependent pathway

**Figure 2:**
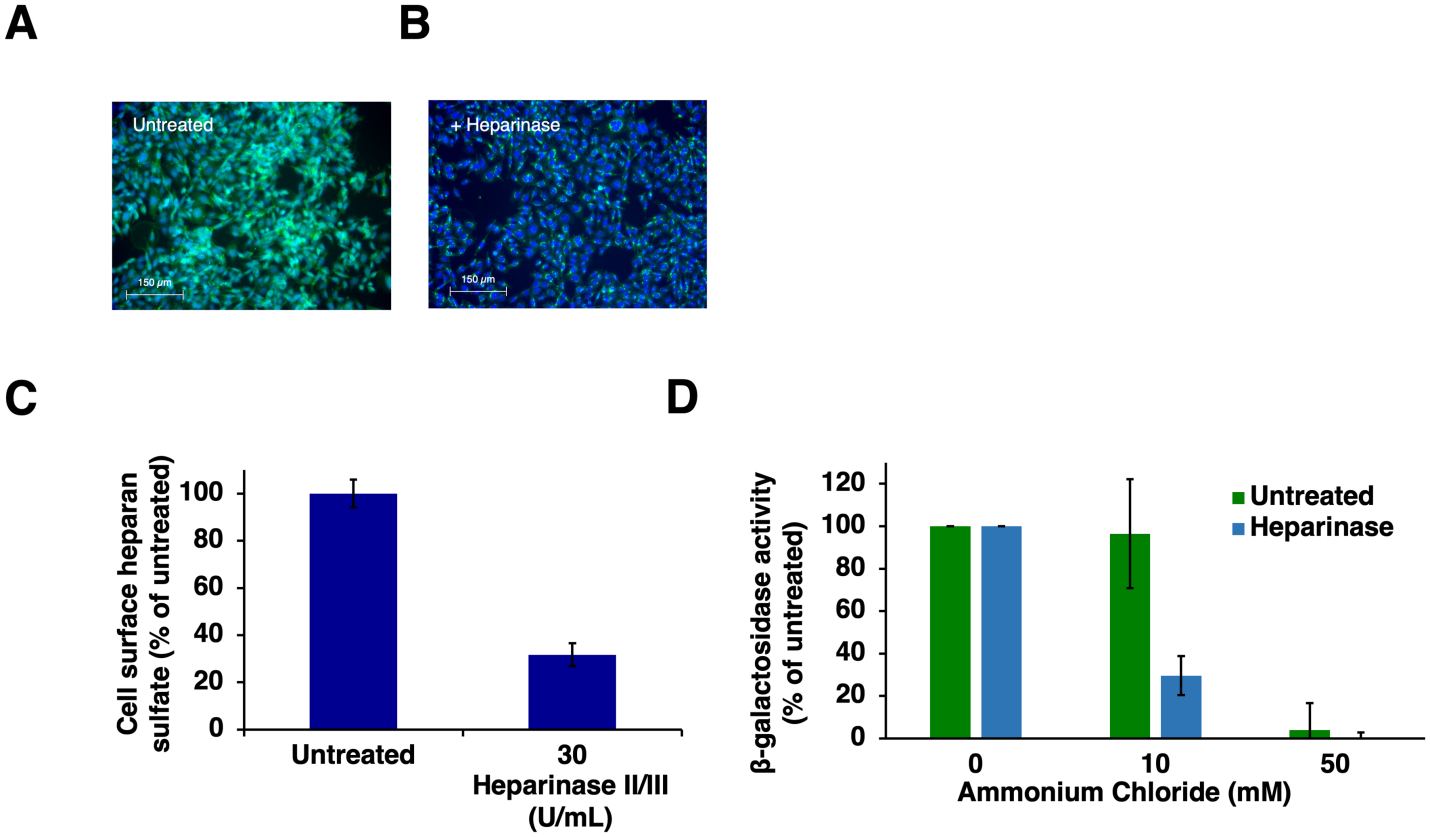
HSV-1 enters heparinase-treated cells by a low pH endosomal pathway. **(A)** CHO-HVEM cells were left untreated or **(B)** treated with heparinase II/III (30 U/mL) for 2 h at 37°C followed by 30 min on ice at 4°C or **(B)**. Cell surface HS was visualized via immunofluorescence microscopy with MAb 10E4 to heparan sulfate **(A,B)**. HS depletion was quantitated with ImageJ **(C)**. **(D)** HSV-1 KOS was added to untreated or heparinase-treated CHO-HVEM cells (MOI of 1) for 6 h. Entry was quantitated as β-galactosidase activity. Results are the mean of three independent experiments with standard error.

Entering HSV-1 can travel multiple routes into a host cell. Selection of HSV-1 entry pathway involves both viral and host factors and is incompletely understood (Chowdhury et al., 2013; Stiles et al., 2008; Roller et al., 2008; Gianni et al., 2010; Gianni et al., 2004; Arii et al., 2009; Nicola and Straus, 2004; Milne et al., 2005; Komala Sari et al., 2013; Delboy et al., 2006; Komala Sari et al., 2020c). HSV-1 interaction with cell surface HS is the very first interaction of HSV-1 with its host cell, yet its contribution to entry pathway has not been investigated previously. Since gC is selectively important for low pH entry and is a viral ligand for heparan sulfate, we determined the role of cell surface HS in HSV-1 entry via a low pH pathway. HS was enzymatically removed from CHO-HVEM cell surfaces by treatment with heparinases, and then entry in the presence of ammonium chloride was quantitated. Entry was measured by the beta-galactosidase reporter assay, which is standard in the field. CHO-HVEM cells harbor the lacZ gene under the control of an HSV-1-inducible promoter. Heparinase treatment reduced cell surface levels of HS (Figures 2A–C). Wild-type HSV-1 strain KOS was added to CHO-HVEM cells treated with heparinase or to control-treated cells. Ammonium chloride inhibited HSV-1 entry into both the HS-deficient and untreated cells, indicative of HSV-1 traveling a low pH, endocytic pathway to infect these cells (Figure 2D). These results suggest that cell surface HS does not direct HSV-1 to the low pH endosomal pathway taken in CHO-HVEM cells. It is possible that HS may determine the entry pathway in the context of other cell types.

### The entry pathway of HSV-1 is not altered by the HS-binding activity of virions

**Figure 3:**
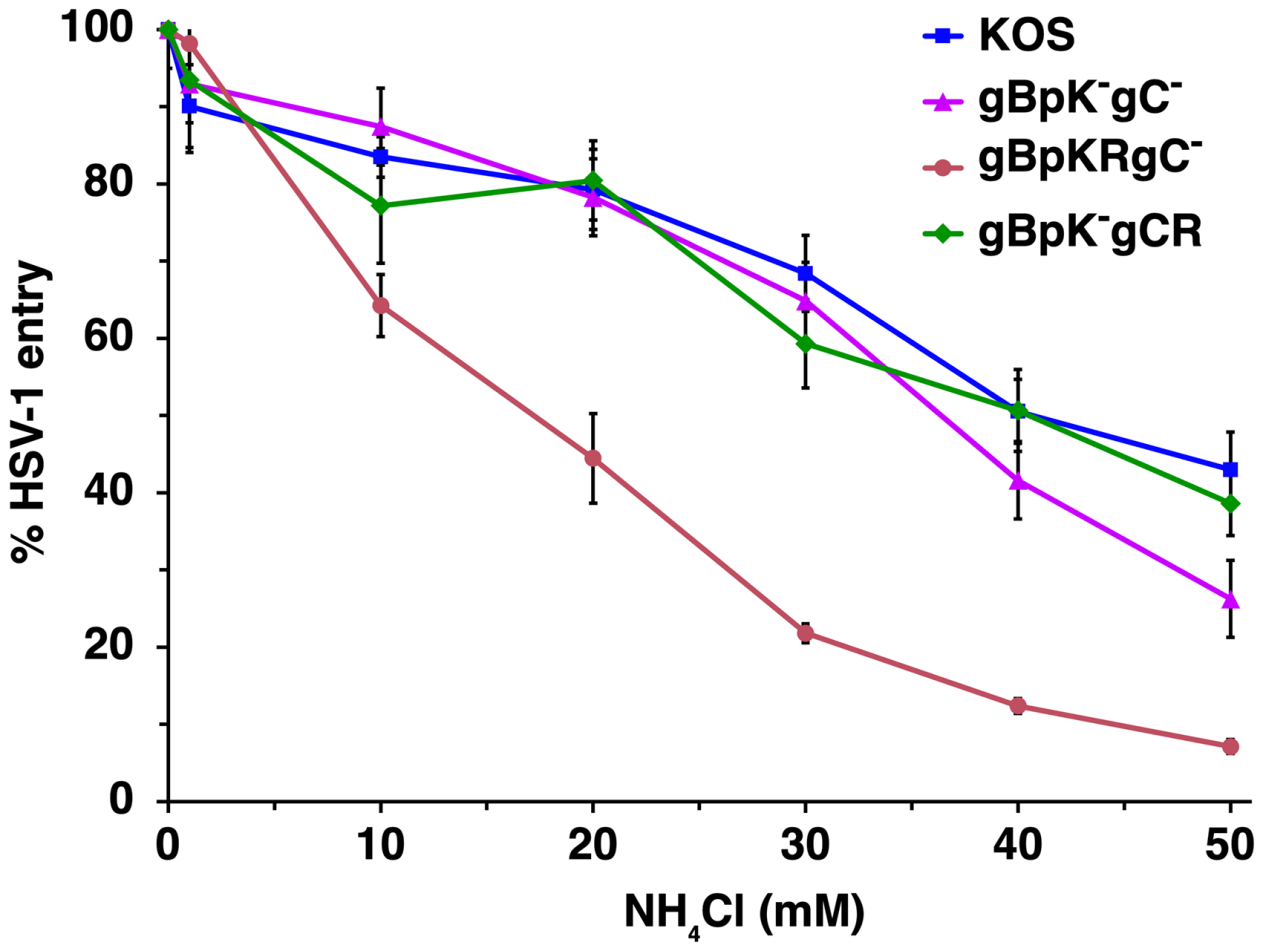
HSV-1 enters cells via a low pH endosomal pathway regardless of its HS-binding capability. Wild type HSV-1 KOS (blue), HSV-1 gBpK^−^gCR, which lacks the pK region of gB (pink), HSV-1 gB pKRgC^−^, which lacks gC (red) or HSV-1 gBpK^−^gC^−^, which lacks both gC and the pK region of gB (green) were added to CHO-HVEM cells (MOI ~ 0.2–0.9) in the presence of increasing concentrations of ammonium chloride. At 8 h p.i., entry was quantitated via β-galactosidase reporter assay. Results are the mean of three independent experiments with standard deviation.

HSV-1 gC is the envelope component primarily responsible for HSV-1 attachment to HS during viral entry. HSV-1 gB contains a lysine-rich (pK) sequence (amino acids 68 to 76 in domain VI near the N-terminus of gB) that also contributes to HSV-1 binding to HS (Laquerre et al., 1998). We employed virions deleted for one or more of the HS binding components to determine their contribution to the route of entry, which has not been assessed previously. Wild-type or mutant HSV-1 s were added to CHO-HVEM cells treated with ammonium chloride, and viral entry was measured by the β-galactosidase reporter assay. Ammonium chloride treatment resulted in a concentration-dependent inhibition of the entry of wild type HSV-1 strain KOS, HSV-1 gBpK^−^gCR, which lacks the pK region of gB, HSV-1 gB pKRgC^−^, which lacks gC, and HSV-1 gBpK^−^gC^−^, which lacks both gC and the pK region of gB (Figure 3). Entry of all viruses was inhibited by >60% at the highest concentration tested, suggesting that entry was low pH-dependent in all cases. HSV-1 entry that is only inhibited by 20% or less by ammonium chloride is considered pH-independent or pH-neutral (Nicola et al., 2005; Nicola et al., 2003; Delboy et al., 2006). These results suggest that the ability of HSV-1 to attach to HS does not contribute to the subsequent selection of viral entry pathway into CHO-HVEM cells.

### gC influences HSV-1 entry at a step downstream of endocytic internalization of virions from the plasma membrane

**Figure 4:**
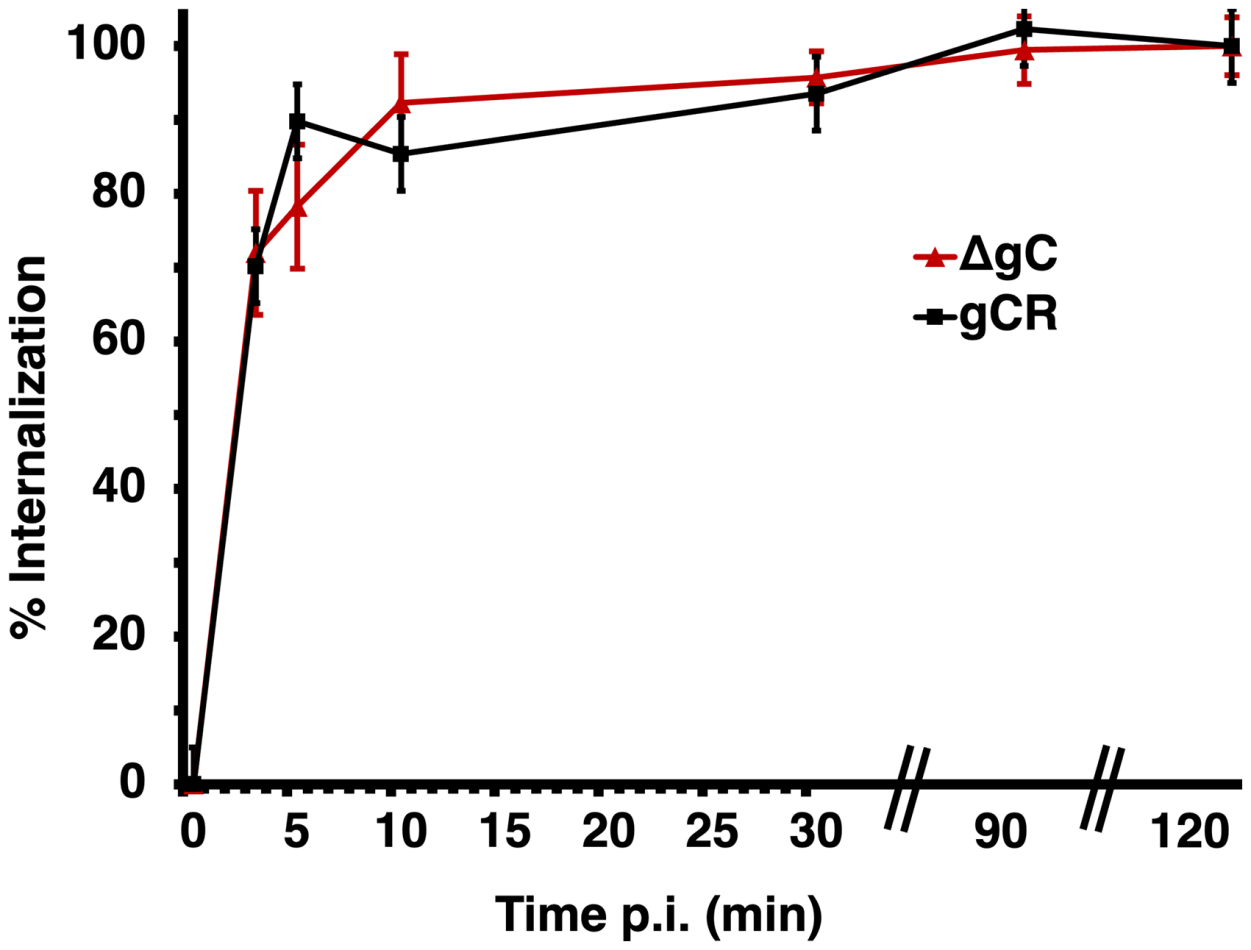
Role of gC in endocytic internalization of HSV-1 from the plasma membrane. HSV-1 ΔgC or gCR was added to CHO-HVEM cells on ice at 4°C for 1 h. After shift to 37°C, extracellular virus was inactivated with sodium citrate at the indicated times p.i. At 6 h p.i., entry was detected by quantitating HSV-1 ICP4-positive cells by immunofluorescence microscopy. Mean and standard deviation of three independent experiments are shown.

The selective role of gC during endocytic entry does not occur at the step of attachment to the cell surface (Komala Sari et al., 2020c). The virus-cell interactions responsible for HSV-1 cell attachment and receptor binding have been well-studied. The mechanism of virion internalization from the cell surface into a host cell vesicular compartment is less clear, including the role of gC, which has not been investigated. To assess the role that gC plays in the initial endocytic internalization from the plasma membrane, we examined surface uptake of HSV-1 ΔgC in CHO-HVEM cells, which support HSV-1 entry by endocytosis. The kinetics of endocytic uptake of ΔgC and wild type gCR were rapid and very similar (Figure 4), suggesting that gC does not contribute to the endocytic internalization mechanism in these cells. Previous results indicated that gC does not affect virus attachment to the cell surface under similar experimental conditions (Komala Sari et al., 2020c). However, gC does facilitate HSV penetration of endosomes (Figures 1B,C; Komala Sari et al., 2020c), and low pH-triggered changes in gB (Dollery et al., 2010), both of which occur downstream of internalization. Taken together, the results to date support a model whereby gC functions at an entry step that occurs after both HSV-1 attachment and endocytic uptake from the plasma membrane.

### Effect of gC on HSV-1 glycoprotein-mediated cell–cell fusion

**Figure 5:**
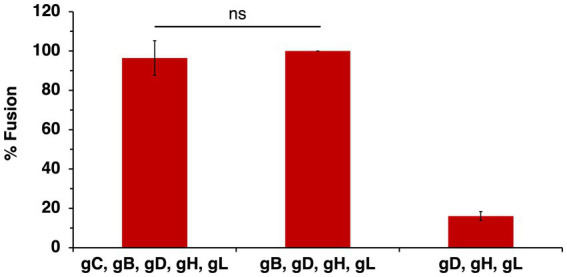
Effect of gC on wild type HSV-1 glycoprotein-mediated cell–cell fusion. CHO-K1 effector cells expressing HSV-1 KOS gB, gD, gH, gL, and T7 polymerase with or without gC were co-cultured for 18 h with CHO-HVEM target cells transfected with the luciferase plasmid. Cell–cell fusion activity of KOS gB, gD, gH, and gL was set to 100%. Results are the averages of four independent experiments. ns, not significant, Student’s *t*-test.

gC facilitates penetration in cells that support endocytic entry (Komala Sari et al., 2020c); Figures 1B,C. Moreover, gC regulates gB fusion function by optimizing gB conformational changes (Komala Sari et al., 2020c). Penetration likely coincides with fusion of the HSV-1 envelope with the limiting membrane of an intracellular compartment during entry into a subset of cell types. HSV-1 entry, assembly and spread all require membrane fusion events. The execution and regulation of these processes require distinct yet overlapping sets of viral proteins and host cell factors (Weed and Nicola, 2017). HSV-1-cell fusion during entry is difficult to study in a direct, reliable, and quantitative manner. Fusion activity of enveloped viruses is routinely assessed with virus-free cell–cell fusion reporter assays. Cell–cell fusion activity has long been considered a surrogate assay for HSV-1-cell fusion during entry, and current models of the HSV-1 fusion mechanism are based largely on results from cell–cell fusion assays. Effector cells are transiently transfected with viral glycoproteins to mimic the virus and are mixed with target cells that bear host cell entry receptors. T7 RNA polymerase is expressed in effector cells and a luciferase reporter gene under control of the T7 promoter is expressed in target cells. The two cell populations are co-cultivated and luciferase activity is measured as an indicator of fusion. gB, gD, and gH/gL on the effector membrane are necessary and sufficient for HSV-1 cell–cell fusion (Turner et al., 1998; Muggeridge, 2000). Importantly, the impact of gC on HSV-1 cell–cell fusion has not been assessed previously. Effector CHO cells transiently expressing wild type KOS strain gB, gD, and gH/gL were added to target CHO-HVEM cells, resulting in robust cell–cell fusion. The presence of gC in the effector cell resulted in little effect on cell–cell fusion (Figure 5), suggesting that gC did not impact the HSV-1 fusion activity measured in this assay. Notably, envelope glycoproteins gM/gN, or gK decrease HSV-1 cell–cell fusion mediated by gB, gD, and gH/gL (Crump et al., 2004; Avitabile et al., 2003). Virion gC influences HSV-1 penetration into cells (virus-cell fusion) in the presence of endosomal low pH. HSV-1 cell–cell fusion occurs at physiological pH and does not require addition of low pH buffer to the cells, as is required, for example, for influenza HA-mediated cell–cell fusion. Virus entry and cell–cell fusion results do not always correlate, as they are quite different processes involving distinct effector and target membranes.

**Figure 6:**
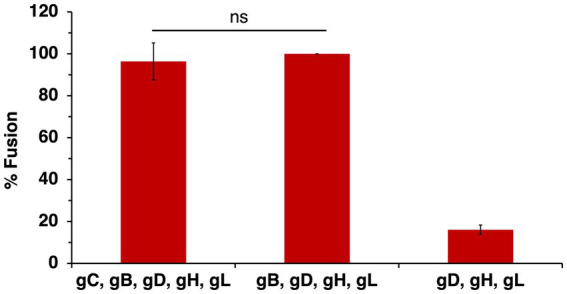
HSV-1 ANG gC does not enhance ANG glycoprotein-mediated cell–cell fusion. CHO-K1 effector cells expressing HSV-1 ANG gB, gD, gH, gL, and T7 polymerase with or without ANG gC were co-cultured for 18 h with CHO-nectin-2 cells transfected with the luciferase plasmid. Cell–cell fusion activity of ANG gD, gB, gH, and gL was set to 100%. Results are presented as the average of three independent experiments. ns, not significant, Student’s *t*-test.

The HSV-1 strain ANG is hyperfusogenic and distinct from most wild type strains. ANG forms syncytia, has fusion-from-without activity, enters cells at reduced temperatures, and has elevated cell–cell fusion activity (Falke et al., 1985; Lingen et al., 1995; Gianopulos et al., 2024). Sequencing revealed multiple mutations in HSV-1 ANG envelope glycoproteins, including gC, which has five amino acid substitutions relative to wild type strains. ANG gB, gD, and gH/gL are necessary and sufficient for cell–cell fusion (Gianopulos et al., 2024). HSV-1 ANG utilizes nectin-2 as a receptor, leading to fusion and entry (Warner et al., 1998). HSV-1 ANG enters CHO-nectin-2 cells by fusion with the cell surface, in contrast to other wild type strains, which enter CHO cells via endocytosis (Delboy et al., 2006; Roller et al., 2008). All together the hyperfusogenic ANG strain provides a useful and unique platform to dissect the complex nature of HSV-1 fusion and entry. To evaluate the potential of ANG gC in cell–cell fusion, cells expressing ANG gB, gD, and gH/gL together with ANG gC were added to target cells bearing nectin-2. The required ANG glycoproteins plus ANG gC mediated robust cell–cell fusion, similar to that in the absence of gC (Figure 6). These results suggest that despite its mutations, ANG gC does not influence the level of cell–cell fusion.

### Heparin inhibition of nectin-1-mediated cell–cell fusion in the presence of gC is independent of cell surface heparan sulfate

**Figure 7:**
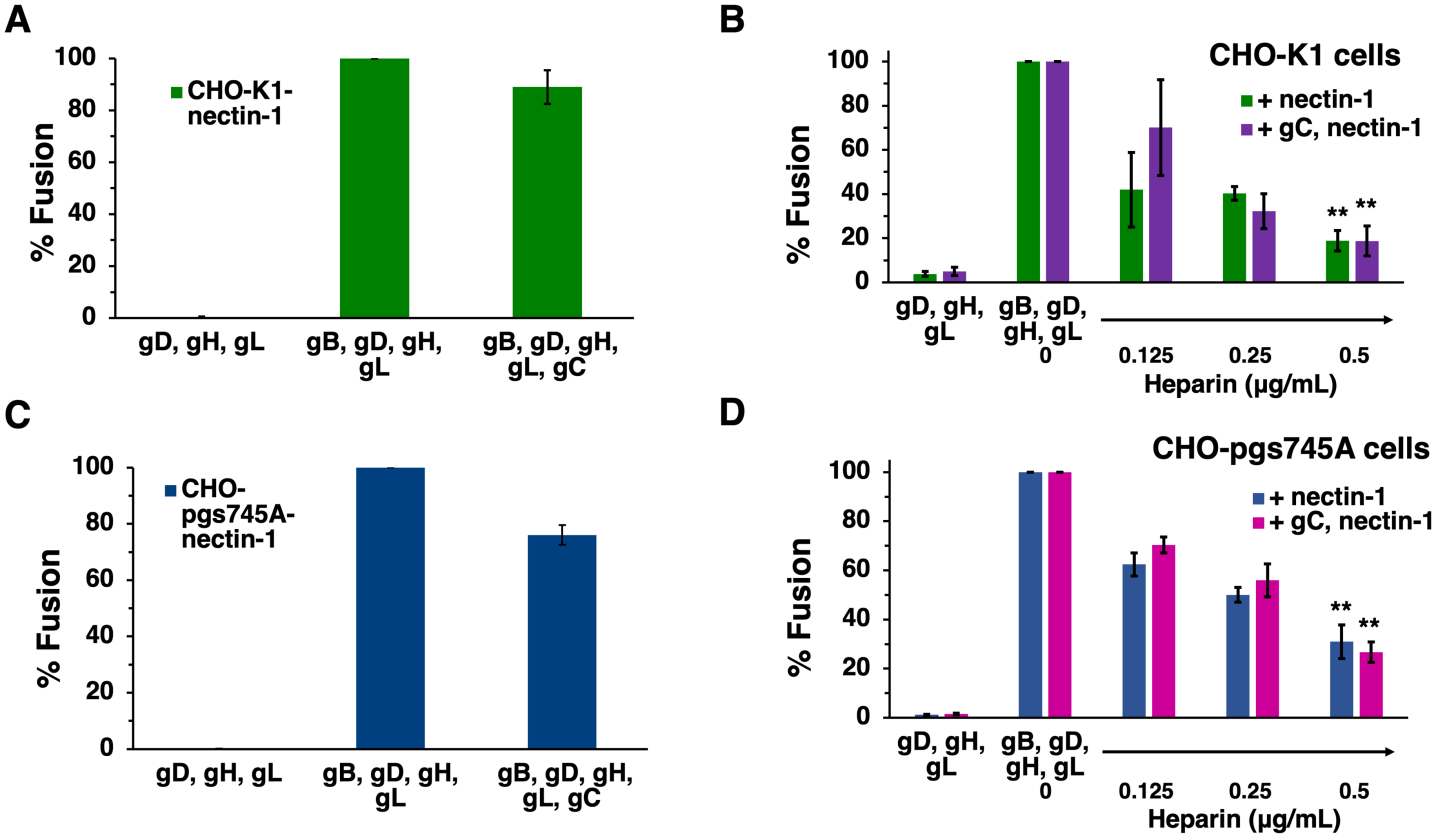
Soluble heparin inhibits nectin-1-mediated cell–cell fusion in the presence and absence of gC. CHO-K1 **(A,B)** or CHO-pgs745A **(C,D)** effector cells expressing gB, gD, gH, gL, and T7 polymerase were co-cultured for 18 h with target cells of the same type transfected with nectin-1 and the luciferase plasmid. Glycoprotein C was transfected into effector cells where indicated. Soluble heparin was added at the time of co-culture **(B,D)**. Cell–cell fusion activity without gC **(A,C)** or heparin **(B,D)** present was set to 100%. Results are averages of three independent experiments. **(B,D)** **p-value < 0.01, Student’s *t* test comparing 0–0.5 ug/ml heparin.

The presence of HSV-1 gC on the effector membrane does not alter cell–cell fusion mediated by HVEM (Figure 5). To test the impact of gC on fusion mediated by the nectin-1 receptor, CHO-K1 target cells were transfected with nectin-1 and luciferase genes. CHO-K1 effector cells were transfected with gB, gD, gH, gL, gC and T7 polymerase genes. Including gC on the effector membrane had little to no effect on nectin-1-mediated cell–cell fusion (Figure 7A). This experiment was next executed with CHO-pgs745A cells, which are unable to synthesize HS (Esko et al., 1985). CHO-pgs745A cells are defective for efficient attachment of HSV-1 (Shieh et al., 1992). gC on the effector cell membrane had little impact on fusion activity of the HS-deficient cells (Figure 7C).

Heparin is a long, negatively charged, sulfated polysaccharide resembling HS in structure and widely used as an anticoagulant. Because of its solubility and structural similarity, heparin is commonly used to experimentally mimic HS. Heparin inhibits HSV-1 attachment to cells because heparin competes with surface-bound HS for binding to virion gC (WuDunn and Spear, 1989). The presence of HS on cells does not influence cell–cell fusion (Pertel et al., 2001; Browne et al., 2001). However, heparin inhibits HSV-1 cell–cell fusion by a mechanism that is unclear (Foster et al., 2001). Understanding how soluble heparin impairs HSV-1 fusion will lead to a better understanding of the fusion reaction. We investigated the impact of gC and HS on heparin inhibition of cell–cell fusion. Soluble heparin inhibited fusion mediated by gB, gD, and gH/gL in a concentration-dependent manner (Figure 7B), as expected. When gC was present on effector cells, soluble heparin inhibited cell–cell fusion to a similar extent (Figure 7B). Heparin inhibited HSV-1 cell–cell fusion when HS-deficient effector and target cells were tested (Figure 7D). Similar heparin inhibition was detected in the presence or absence of gC (Figure 7D). Together, the results suggest that heparin inhibits cell–cell fusion in the presence of gC on the effector membrane and in the presence of HS on the target and effector cells. This is consistent with a mechanism of heparin inhibition of cell–cell fusion that is independent of soluble heparin competing with cell-associated HS for binding to HSV-1 entry glycoproteins, including gC.

## Discussion

HSV-1 gC functions in endosomal entry independent of its role in virion attachment to the cell surface. gC regulates fusion-associated conformational changes in gB, but the precise mechanism by which gC selectively acts during low pH entry of HSV-1 is not well-understood. Since both gC and gB bind to heparan sulfate (HS), we also probed a potential post-attachment function for HS in fusion and low pH entry. Here we show that HSV-1 lacking gC accumulates in endosomes during viral entry. Further, HS is not a determinant of HSV-1 entry pathway, nor does HS enhance pH-neutral fusion in a cell–cell fusion assay in the presence of gC. Notably, gC-null HSV-1 is rapidly internalized by endocytosis, in a manner similar to wild type HSV-1. The results fill several knowledge gaps in our understanding of gC and HS in HSV-1 entry. Overall, this report supports a post-attachment, post-internalization function for gC in HSV-1 entry by endocytosis that is independent of HS.

HSV-1 gC is a multi-functional glycoprotein present in the viral envelope and on the surface of infected cells. In addition to viral entry functions, gC has immune evasion features and is a component of candidate vaccines (Friedman et al., 1984; Komala Sari et al., 2020b; Hull et al., 2025; Awasthi et al., 2019; Weir et al., 1989). HSV-1 gC, as well as host heparanase activity, facilitates HSV-1 release from infected cells (Hadigal et al., 2015; Frost et al., 2024).

gC is needed for optimal HSV-1 infectivity of cells that support an endosomal entry pathway such as primary human epidermal keratinocytes and model CHO-receptor cells (Komala Sari et al., 2020c). The experimental design in this previous report permitted the conclusion that gC’s impact on endocytic entry is independent of its function in virion attachment. Enveloped virions that lack gC undergo prolonged endosomal trafficking during entry by endocytosis (Komala Sari et al., 2020c) and results here visually document accumulation in endocytic vesicles (Figures 1B,C). The anti-gC MAb 3G9 targets the gC N-terminus and selectively inhibits HSV-1 entry via a low pH pathway (Gianopulos et al., 2022). In the absence of gC, fusion-associated conformational changes in gB require an even lower pH, suggesting that gC positively regulates gB’s fusion function (Komala Sari et al., 2020c). gC itself undergoes low pH-triggered conformational changes. Changes in gC’s antigenic structure occur at pH < 6 (Gianopulos et al., 2022). Conformational changes in gB are triggered at a similar mildly acidic pH threshold (Dollery et al., 2010; Siekavizza-Robles et al., 2010; Dollery et al., 2011; Wudiri et al., 2017; Weed et al., 2018; Komala Sari et al., 2020a). The changes in gC are reversible, as they are in gB. Thus, the coordinated action of gC and gB may result in optimal fusion activity during low pH entry. Direct interactions between gB and gC are difficult to detect. A viral or cellular molecule other than HS might serve as an intermediary between gB and gC during endosomal entry of HSV-1. Based on the data presented here and in previous reports, we propose a model whereby gC promotes penetration from the low pH endosomes of epithelial cells by optimizing fusion-associated conformational changes in gB. The impact of gC on this process is independent of its interaction with heparan sulfate. Future work is needed to confirm and extend gC’s mechanism of action in endocytic entry.

Cell surface glycosaminoglycans including heparan sulfate are primary attachment receptors for many viruses, including members of the *Herpesviridae*. HS does not participate in the HSV-1 fusion reaction but serves to initially attract virions to the cells facilitating subsequent interaction with required entry receptors such as nectin-1 or HVEM (Shukla and Spear, 2001). HS itself or the ability of virions to bind to HS do not help direct incoming HSV-1 particles to a low pH pathway (Figures 2, 3).

Cellular HS is not necessary for transfected cell–cell fusion mediated by gB, gD, and gH/gL. Spinoculation of entry glycoprotein-expressing CHO-K1 cells does not increase fusion, suggesting proximity between cells, which could be facilitated by heparan sulfate, is not a factor in cell–cell fusion (Scanlan et al., 2005). HS-deficient CHO-pgs745A cells do not exhibit significantly different fusion properties compared to wild type cells in the presence of gC (Figure 7C). This suggests that even when the primary HS-binding HSV-1 glycoprotein is present, HS on the cell surface does not contribute to cell–cell fusion. gC does not affect transfected cell fusion mediated by the four HSV-1 fusion glycoproteins (Figure 5). Importantly, this does not preclude a function for gC in low pH fusion. We have maintained that HSV-1 harbors the capacity to execute both pH-neutral and low pH-triggered membrane fusion. There is much evidence that low pH is an important factor in HSV-1 entry. It is well-established that alphaherpesvirus entry requires endosomal low pH in a cell-specific manner (Nicola, 2016; Pastenkos et al., 2018; Miller et al., 2019). Mildly acidic pH-treatment inactivates HSV-1 particles in isolation, a hallmark of viruses that fuse at low pH, and gB is the target of inactivation (Nicola et al., 2003; Weed et al., 2017). Low pH triggers binding of HSV-1 to liposomes containing HVEM and detectable *in vitro* fusion of HSV-1 and liposomes (Whitbeck et al., 2006; Ramirez et al., 2023). This report contributes to the mounting indirect evidence that gC may facilitate pH-triggered fusion during HSV-1 entry and penetration from the endosome.

Soluble heparin inhibits fusion mediated by gB, gD, and gH/gL in the virus-free, cell–cell assays (Foster et al., 2001). The presence of gC does not alter heparin inhibition of cell–cell fusion (Figure 7B). Mutations in the C-terminal tail domain of gB counteract the inhibitory effects of soluble heparin on cell–cell fusion (Foster et al., 2001). Heparin inhibits HSV-1 cell–cell fusion regardless of the presence of HS or gC (Figures 7B,D). Soluble heparin increases HSV-1 syncytium formation, a related yet distinct HSV-1 fusion process whereby infected cells fuse with uninfected cells (Shieh and Spear, 1994). All together, our results suggest that the mechanism underlying inhibition of cell–cell fusion by heparin does not involve competitive binding between HS and soluble heparin for cell-associated gC or gB. The gB and gH cytoplasmic tails are proposed to functionally interact for fusion to occur (Rogalin and Heldwein, 2015). It remains to be determined whether soluble heparin interaction with glycoprotein ectodomains blocks gB-gH interactions necessary for fusion.

Evidence is provided here that gC facilitates HSV-1 penetration following endocytic uptake. gC does not contribute to pH-neutral cell–cell fusion; thus, a robust, direct assay of low pH fusion of HSV-1 particles with the host endosomal membrane would shed further light on this novel function of gC. Our current model of endocytic entry of HSV-1 is that gC positively regulates conformational changes in gB resulting in low pH-triggered fusion of virus and endosome membranes.

## Data Availability

The raw data supporting the conclusions of this article will be made available by the authors, without undue reservation.

## References

[ref1] Arii J. Uema M. Morimoto T. Sagara H. Akashi H. Ono E.. (2009). Entry of herpes simplex virus 1 and other alphaherpesviruses via the paired immunoglobulin-like type 2 receptor alpha. J. Virol. 83, 4520–4527. doi: 10.1128/JVI.02601-08 19244335 PMC2668467

[ref2] Avitabile E. Lombardi G. Campadelli-Fiume G. (2003). Herpes simplex virus glycoprotein K, but not its syncytial allele, inhibits cell-cell fusion mediated by the four fusogenic glycoproteins, gD, gB, gH, and gL. J. Virol. 77, 6836–6844. doi: 10.1128/JVI.77.12.6836-6844.2003, PMID: 12768003 PMC156197

[ref3] Awasthi S. Hook L. M. Pardi N. Wang F. Miles A. Cancro M. P.. (2019). Nucleoside-modified mrna encoding Hsv-2 glycoproteins C, D, and E prevents clinical and subclinical genital herpes. Sci. Immunol. 20:eaaw7083. doi: 10.1126/sciimmunol.aaw7083 PMC6822172 31541030

[ref4] Browne H. Bruun B. Minson T. (2001). Plasma membrane requirements for cell fusion induced by herpes simplex virus type 1 glycoproteins gB, gD, gH and gL. J. Gen. Virol. 82, 1419–1422. doi: 10.1099/0022-1317-82-6-1419, PMID: 11369886

[ref5] Chowdhury S. Chouljenko V. N. Naderi M. Kousoulas K. G. (2013). The amino terminus of herpes simplex virus 1 glycoprotein K is required for virion entry via the paired immunoglobulin-like type-2 receptor alpha. J. Virol. 87, 3305–3313. doi: 10.1128/JVI.02982-12, PMID: 23302878 PMC3592154

[ref6] Connolly S. A. Jardetzky T. S. Longnecker R. (2020). The structural basis of herpesvirus entry. Nat. Rev. Microbiol. 19, 110–121. doi: 10.1038/s41579-020-00448-w, PMID: 33087881 PMC8579738

[ref7] Crump C. M. Bruun B. Bell S. Pomeranz L. E. Minson T. Browne H. M. (2004). Alphaherpesvirus glycoprotein M causes the relocalization of plasma membrane proteins. J. Gen. Virol. 85, 3517–3527. doi: 10.1099/vir.0.80361-0, PMID: 15557225

[ref8] Delboy M. G. Patterson J. L. Hollander A. M. Nicola A. V. (2006). Nectin-2-mediated entry of a syncytial strain of herpes simplex virus via pH-independent fusion with the plasma membrane of Chinese hamster ovary cells. Virol. J. 3:105. doi: 10.1186/1743-422X-3-105, PMID: 17192179 PMC1779275

[ref9] Dollery S. J. Delboy M. G. Nicola A. V. (2010). Low pH-induced conformational change in herpes simplex virus glycoprotein B. J. Virol. 84, 3759–3766. doi: 10.1128/JVI.02573-09, PMID: 20147407 PMC2849479

[ref10] Dollery S. J. Wright C. C. Johnson D. C. Nicola A. V. (2011). Low-pH-dependent changes in the conformation and oligomeric state of the prefusion form of herpes simplex virus glycoprotein B are separable from fusion activity. J. Virol. 85, 9964–9973. doi: 10.1128/JVI.05291-11, PMID: 21813610 PMC3196434

[ref11] Esko J. D. Stewart T. E. Taylor W. H. (1985). Animal cell mutants defective in glycosaminoglycan biosynthesis. Proc. Natl. Acad. Sci. USA 82, 3197–3201, PMID: 3858816 10.1073/pnas.82.10.3197 PMC397742

[ref12] Falke D. Knoblich A. Muller S. (1985). Fusion from without induced by herpes simplex virus type 1. Intervirology 24, 211–219. doi: 10.1159/000149645 3000981

[ref13] Foster T. P. Melancon J. M. Kousoulas K. G. (2001). An alpha-helical domain within the carboxyl terminus of herpes simplex virus type 1 (Hsv-1) glycoprotein B (gB) is associated with cell fusion and resistance to heparin inhibition of cell fusion. Virology 287, 18–29. doi: 10.1006/viro.2001.1004, PMID: 11504538

[ref14] Friedman H. M. Cohen G. H. Eisenberg R. J. Seidel C. A. Cines D. B. (1984). Glycoprotein C of herpes simplex virus 1 acts as a receptor for the C3b complement component on infected cells. Nature 309, 633–635. doi: 10.1038/309633a0, PMID: 6328323

[ref15] Frost T. C. Salnikov M. Rice S. A. (2024). Enhancement of Hsv-1 cell-free virion release by the envelope protein gC. Virology 596:110120. doi: 10.1016/j.virol.2024.110120, PMID: 38805801 PMC11178091

[ref16] Fuller A. O. Spear P. G. (1987). Anti-glycoprotein D antibodies that permit adsorption but block infection by herpes simplex virus 1 prevent virion-cell fusion at the cell surface. Proc. Natl. Acad. Sci. USA 84, 5454–5458, PMID: 3037552 10.1073/pnas.84.15.5454 PMC298876

[ref17] Gianni T. Campadelli-Fiume G. Menotti L. (2004). Entry of herpes simplex virus mediated by chimeric forms of nectin1 retargeted to endosomes or to lipid rafts occurs through acidic endosomes. J. Virol. 78, 12268–12276. doi: 10.1128/JVI.78.22.12268-12276.2004, PMID: 15507614 PMC525084

[ref18] Gianni T. Gatta V. Campadelli-Fiume G. (2010). {alpha}V{beta}3-integrin routes herpes simplex virus to an entry pathway dependent on cholesterol-rich lipid rafts and dynamin2. Proc. Natl. Acad. Sci. USA 107, 22260–22265. doi: 10.1073/pnas.1014923108, PMID: 21135248 PMC3009828

[ref19] Gianopulos K. A. Komala Sari T. Weed D. J. Pritchard S. M. Nicola A. V. (2022). Conformational changes in herpes simplex virus glycoprotein C. J. Virol. 96:e0016322. doi: 10.1128/jvi.00163-22 35913218 PMC9400475

[ref20] Gianopulos K. A. Makio A. O. Pritchard S. M. Cunha C. W. Hull M. A. Nicola A. V. (2024). Herpes simplex virus 1 glycoprotein B from a Hyperfusogenic virus mediates enhanced cell-cell fusion. Viruses 16:251. doi: 10.3390/v16020251 38400027 PMC10892784

[ref21] Gonzalez-Del Pino G. L. Heldwein E. E. (2022). Well put together-a guide to accessorizing with the herpesvirus gH/gL complexes. Viruses 14:296. doi: 10.3390/v14020296 35215889 PMC8874593

[ref22] Hadigal S. R. Agelidis A. M. Karasneh G. A. Antoine T. E. Yakoub A. M. Ramani V. C.. (2015). Heparanase is a host enzyme required for herpes simplex virus-1 release from cells. Nat. Commun. 6:6985. doi: 10.1038/ncomms7985 25912399 PMC4413471

[ref23] Herold B. C. Visalli R. J. Susmarski N. Brandt C. R. Spear P. G. (1994). Glycoprotein C-independent binding of herpes simplex virus to cells requires cell surface heparan sulphate and glycoprotein B. J. Gen. Virol. 75, 1211–1222. doi: 10.1099/0022-1317-75-6-1211, PMID: 8207388

[ref24] Herold B. C. Wudunn D. Soltys N. Spear P. G. (1991). Glycoprotein C of herpes simplex virus type 1 plays a principal role in the adsorption of virus to cells and in infectivity. J. Virol. 65, 1090–1098. doi: 10.1128/jvi.65.3.1090-1098.1991, PMID: 1847438 PMC239874

[ref25] Hull M. A. Pritchard S. M. Nicola A. V. (2025). Herpes simplex virus 1 envelope glycoprotein C shields glycoprotein D to protect virions from entry-blocking antibodies. J. Virol. e00090–25. 40135897 10.1128/jvi.00090-25 PMC11998518

[ref26] Hung S. L. Srinivasan S. Friedman H. M. Eisenberg R. J. Cohen G. H. (1992). Structural basis of C3b binding by glycoprotein C of herpes simplex virus. J. Virol. 66, 4013–4027. 1602532 10.1128/jvi.66.7.4013-4027.1992 PMC241204

[ref27] Komala Sari T. Gianopulos K. A. Nicola A. V. (2020a). Conformational change in herpes simplex virus entry glycoproteins detected by dot blot. Methods Mol. Biol. 2060, 319–326. doi: 10.1007/978-1-4939-9814-2_18, PMID: 31617187 PMC9707355

[ref28] Komala Sari T. Gianopulos K. A. Nicola A. V. (2020b). Glycoprotein C of herpes simplex virus 1 shields glycoprotein B from antibody neutralization. J. Virol. 94, e01852–e01819. doi: 10.1128/JVI.01852-19 31826995 PMC7022361

[ref29] Komala Sari T. Gianopulos K. A. Weed D. J. Schneider S. M. Pritchard S. M. Nicola A. V. (2020c). Herpes simplex virus glycoprotein C regulates low pH entry. mSphere 5, e00826–e00819. doi: 10.1128/mSphere.00826-19 32024702 PMC7002311

[ref30] Komala Sari T. Pritchard S. M. Cunha C. W. Wudiri G. A. Laws E. I. Aguilar H. C.. (2013). Contributions of herpes simplex virus 1 envelope proteins to entry by endocytosis. J. Virol. 87, 13922–13926. doi: 10.1128/JVI.02500-13, PMID: 24109213 PMC3838226

[ref31] Koyama A. H. Uchida T. (1987). The mode of entry of herpes simplex virus type 1 into Vero cells. Microbiol. Immunol. 31, 123–130, PMID: 3037281 10.1111/j.1348-0421.1987.tb03075.x

[ref32] Laquerre S. Argnani R. Anderson D. B. Zucchini S. Manservigi R. Glorioso J. C. (1998). Heparan sulfate proteoglycan binding by herpes simplex virus type 1 glycoproteins B and C, which differ in their contributions to virus attachment, penetration, and cell-to-cell spread. J. Virol. 72, 6119–6130, PMID: 9621076 10.1128/jvi.72.7.6119-6130.1998 PMC110418

[ref33] Lingen M. Seck T. Weise K. Falke D. (1995). Single amino acid substitutions in the glycoprotein B carboxy terminus influence the fusion from without property of herpes simplex virus type 1. J. Gen. Virol. 76, 1843–1849. doi: 10.1099/0022-1317-76-7-1843, PMID: 9049391

[ref34] Madavaraju K. Koganti R. Volety I. Yadavalli T. Shukla D. (2020). Herpes simplex virus cell entry mechanisms: an update. Front. Cell. Infect. Microbiol. 10:617578. doi: 10.3389/fcimb.2020.617578 33537244 PMC7848091

[ref35] Miller J. L. Weed D. J. Lee B. H. Pritchard S. M. Nicola A. V. (2019). Low-pH endocytic entry of the porcine Alphaherpesvirus pseudorabies virus. J. Virol. 93:e01849-18. doi: 10.1128/JVI.01849-18 30355685 PMC6321905

[ref36] Milne R. S. Nicola A. V. Whitbeck J. C. Eisenberg R. J. Cohen G. H. (2005). Glycoprotein D receptor-dependent, low-pH-independent endocytic entry of herpes simplex virus type 1. J. Virol. 79, 6655–6663. doi: 10.1128/JVI.79.11.6655-6663.2005, PMID: 15890903 PMC1112142

[ref37] Montgomery R. I. Warner M. S. Lum B. J. Spear P. G. (1996). Herpes simplex virus-1 entry into cells mediated by a novel member of the Tnf/Ngf receptor family. Cell 87, 427–436, PMID: 8898196 10.1016/s0092-8674(00)81363-x

[ref38] Muggeridge M. I. (2000). Characterization of cell-cell fusion mediated by herpes simplex virus 2 glycoproteins gB, gD, gH and gL in transfected cells. J. Gen. Virol. 81, 2017–2027. doi: 10.1099/0022-1317-81-8-2017, PMID: 10900041

[ref39] Nicola A. V. (2016). Herpesvirus entry into host cells mediated by endosomal low pH. Traffic 17, 965–975. doi: 10.1111/tra.12408, PMID: 27126894 PMC5444542

[ref40] Nicola A. V. Hou J. Major E. O. Straus S. E. (2005). Herpes simplex virus type 1 enters human epidermal keratinocytes, but not neurons, via a pH-dependent endocytic pathway. J. Virol. 79, 7609–7616. doi: 10.1128/JVI.79.12.7609-7616.2005, PMID: 15919913 PMC1143659

[ref41] Nicola A. V. Mcevoy A. M. Straus S. E. (2003). Roles for endocytosis and low pH in herpes simplex virus entry into HeLa and Chinese hamster ovary cells. J. Virol. 77, 5324–5332. doi: 10.1128/jvi.77.9.5324-5332.2003, PMID: 12692234 PMC153978

[ref42] Nicola A. V. Straus S. E. (2004). Cellular and viral requirements for rapid endocytic entry of herpes simplex virus. J. Virol. 78, 7508–7517. doi: 10.1128/JVI.78.14.7508-7517.2004, PMID: 15220424 PMC434080

[ref43] Pastenkos G. Lee B. Pritchard S. M. Nicola A. V. (2018). Bovine herpesvirus 1 entry by a low-pH endosomal pathway. J. Virol. 92, e00839–e00818. doi: 10.1128/JVI.00839-18 30045989 PMC6158438

[ref44] Pertel P. E. Fridberg A. Parish M. L. Spear P. G. (2001). Cell fusion induced by herpes simplex virus glycoproteins gB, gD, and gH-gL requires a gD receptor but not necessarily heparan sulfate. Virology 279, 313–324. doi: 10.1006/viro.2000.0713 11145912

[ref45] Ramirez J. M. Calderon-Zavala A. C. Balaram A. Heldwein E. E. (2023). *In vitro* reconstitution of herpes simplex virus 1 fusion identifies low pH as a fusion co-trigger. MBio 14:e0208723. doi: 10.1128/mbio.02087-23 37874146 PMC10746285

[ref46] Rogalin H. B. Heldwein E. E. (2015). Interplay between the herpes simplex virus 1 gB Cytodomain and the gH Cytotail during cell-cell fusion. J. Virol. 89, 12262–12272. doi: 10.1128/JVI.02391-15, PMID: 26401042 PMC4665236

[ref47] Roller D. G. Dollery S. J. Doyle J. L. Nicola A. V. (2008). Structure-function analysis of herpes simplex virus glycoprotein B with fusion-from-without activity. Virology 382, 207–216. doi: 10.1016/j.virol.2008.09.015 18950828

[ref48] Scanlan P. M. Tiwari V. Bommireddy S. Shukla D. (2005). Spinoculation of heparan sulfate deficient cells enhances Hsv-1 entry, but does not abolish the need for essential glycoproteins in viral fusion. J. Virol. Methods 128, 104–112. doi: 10.1016/j.jviromet.2005.04.008, PMID: 15908019

[ref49] Sears A. E. Mcgwire B. S. Roizman B. (1991). Infection of polarized Mdck cells with herpes simplex virus 1: two asymmetrically distributed cell receptors interact with different viral proteins. Proc. Natl. Acad. Sci. USA 88, 5087–5091, PMID: 1647025 10.1073/pnas.88.12.5087 PMC51816

[ref50] Shieh M. T. Spear P. G. (1994). Herpesvirus-induced cell fusion that is dependent on cell surface heparan sulfate or soluble heparin. J. Virol. 68, 1224–1228. doi: 10.1128/jvi.68.2.1224-1228.1994, PMID: 8289356 PMC236566

[ref51] Shieh M. T. Wudunn D. Montgomery R. I. Esko J. D. Spear P. G. (1992). Cell surface receptors for herpes simplex virus are heparan sulfate proteoglycans. J. Cell Biol. 116, 1273–1281. doi: 10.1083/jcb.116.5.1273, PMID: 1310996 PMC2289355

[ref52] Shukla D. Spear P. G. (2001). Herpesviruses and heparan sulfate: an intimate relationship in aid of viral entry. J. Clin. Invest. 108, 503–510. doi: 10.1172/JCI13799 11518721 PMC209412

[ref53] Siekavizza-Robles C. R. Dollery S. J. Nicola A. V. (2010). Reversible conformational change in herpes simplex virus glycoprotein B with fusion-from-without activity is triggered by mildly acidic pH. Virol. J. 7:352. doi: 10.1186/1743-422X-7-352 21122119 PMC3003269

[ref54] Stiles K. M. Milne R. S. Cohen G. H. Eisenberg R. J. Krummenacher C. (2008). The herpes simplex virus receptor nectin-1 is down-regulated after trans-interaction with glycoprotein D. Virology 373, 98–111. doi: 10.1016/j.virol.2007.11.012, PMID: 18076965 PMC2629994

[ref55] Turner A. Bruun B. Minson T. Browne H. (1998). Glycoproteins gB, gD, and gHgL of herpes simplex virus type 1 are necessary and sufficient to mediate membrane fusion in a cos cell transfection system. J. Virol. 72, 873–875, PMID: 9420303 10.1128/jvi.72.1.873-875.1998 PMC109452

[ref56] Warner M. S. Geraghty R. J. Martinez W. M. Montgomery R. I. Whitbeck J. C. Xu R.. (1998). A cell surface protein with herpesvirus entry activity (HveB) confers susceptibility to infection by mutants of herpes simplex virus type 1, herpes simplex virus type 2, and pseudorabies virus. Virology 246, 179–189. doi: 10.1006/viro.1998.9218, PMID: 9657005

[ref57] Weed D. J. Dollery S. J. Komala Sari T. Nicola A. V. (2018). Acidic pH mediates changes in antigenic and Oligomeric conformation of herpes simplex virus gB and is a determinant of cell-specific entry. J. Virol. 92, e01034–e01018. doi: 10.1128/JVI.01034-18, PMID: 29925660 PMC6096812

[ref58] Weed D. J. Nicola A. V. (2017). Herpes simplex virus Membrane Fusion. Adv. Anat. Embryol. Cell Biol. 223, 29–47. doi: 10.1007/978-3-319-53168-7_2, PMID: 28528438 PMC5869023

[ref59] Weed D. J. Pritchard S. M. Gonzalez F. Aguilar H. C. Nicola A. V. (2017). Mildly acidic pH triggers an irreversible conformational change in the fusion domain of herpes simplex virus 1 glycoprotein B and inactivation of viral entry. J. Virol. 91, e02123–e02116. doi: 10.1128/JVI.02123-16, PMID: 28003487 PMC5309949

[ref60] Weir J. P. Bennett M. Allen E. M. Elkins K. L. Martin S. Rouse B. T. (1989). Recombinant vaccinia virus expressing the herpes simplex virus type 1 glycoprotein C protects mice against herpes simplex virus challenge. J. Gen. Virol. 70, 2587–2594. doi: 10.1099/0022-1317-70-10-2587, PMID: 2794972

[ref61] Whitbeck J. C. Zuo Y. Milne R. S. Cohen G. H. Eisenberg R. J. (2006). Stable association of herpes simplex virus with target membranes is triggered by low pH in the presence of the gD receptor, Hvem. J. Virol. 80, 3773–3780. doi: 10.1128/JVI.80.8.3773-3780.2006, PMID: 16571794 PMC1440471

[ref62] Williams R. K. Straus S. E. (1997). Specificity and affinity of binding of herpes simplex virus type 2 glycoprotein B to glycosaminoglycans. J. Virol. 71, 1375–1380. doi: 10.1128/jvi.71.2.1375-1380.1997 8995662 PMC191193

[ref63] Wudiri G. A. Schneider S. M. Nicola A. V. (2017). Herpes simplex virus 1 envelope cholesterol facilitates membrane fusion. Front. Microbiol. 8:2383. doi: 10.3389/fmicb.2017.02383 29270154 PMC5723649

[ref64] Wudunn D. Spear P. G. (1989). Initial interaction of herpes simplex virus with cells is binding to heparan sulfate. J. Virol. 63, 52–58. doi: 10.1128/jvi.63.1.52-58.1989, PMID: 2535752 PMC247656

